# Emerging Contaminants: An Emerging Risk Factor for Diabetes Mellitus

**DOI:** 10.3390/toxics12010047

**Published:** 2024-01-08

**Authors:** Huixia Niu, Manjin Xu, Pengcheng Tu, Yunfeng Xu, Xueqing Li, Mingluan Xing, Zhijian Chen, Xiaofeng Wang, Xiaoming Lou, Lizhi Wu, Shengzhi Sun

**Affiliations:** 1 Zhejiang Provincial Center for Disease Control and Prevention, 3399 Bin Sheng Road, Binjiang District, Hangzhou 310051, China; 2111101061@nbu.edu.cn (H.N.); pchtu@cdc.zj.cn (P.T.); xqli@cdc.zj.cn (X.L.); mlxing@cdc.zj.cn (M.X.); zhjchen@cdc.zj.cn (Z.C.); xfwang@cdc.zj.cn (X.W.); xmlou@cdc.zj.cn (X.L.); 2 School of Public Health, Xiamen University, Xiang’an South Road, Xiang’an District, Xiamen 361102, China; 32620192200571@stu.xmu.edu.cn (M.X.); 32620192200574@stu.xmu.edu.cn (Y.X.); 3 School of Public Health, Capital Medical University, Beijing 100069, China

**Keywords:** emerging contaminants diabetes mellitus, gut microbiota, dietary exposure, emerging pollutants

## Abstract

Emerging contaminants have been increasingly recognized as critical determinants in global public health outcomes. However, the intricate relationship between these contaminants and glucose metabolism remains to be fully elucidated. The paucity of comprehensive clinical data, coupled with the need for in-depth mechanistic investigations, underscores the urgency to decipher the precise molecular and cellular pathways through which these contaminants potentially mediate the initiation and progression of diabetes mellitus. A profound understanding of the epidemiological impact of these emerging contaminants, as well as the elucidation of the underlying mechanistic pathways, is indispensable for the formulation of evidence-based policy and preventive interventions. This review systematically aggregates contemporary findings from epidemiological investigations and delves into the mechanistic correlates that tether exposure to emerging contaminants, including endocrine disruptors, perfluorinated compounds, microplastics, and antibiotics, to glycemic dysregulation. A nuanced exploration is undertaken focusing on potential dietary sources and the consequential role of the gut microbiome in their toxic effects. This review endeavors to provide a foundational reference for future investigations into the complex interplay between emerging contaminants and diabetes mellitus.

## 1. Introduction

According to the International Diabetes Federation, the global number of individuals with diabetes reached 425 million in 2017 [1]. Projections suggest that by 2045, the global diabetic population will escalate to 783 million. Diabetes is understood to be a polygenic hereditary disorder with a pronounced genetic predisposition. Approximately 60% of type II diabetes patients have a familial history of the disease, showcasing a notable familial aggregation [2]. Current genome-wide association studies have pinpointed over 80 susceptibility loci associated with diabetes [3]. Beyond genetic predispositions, environmental factors exert an undeniable influence on diabetes outcomes [4], with emerging pollutants increasingly taken into consideration [5].

With the advent of modern industrial developments and the introduction of novel chemical compounds, the spectrum of environmental pollutants has expanded. Many of these emerging contaminants possess chemical and toxicological properties that remain insufficiently characterized. These pollutants emanate from diverse sources, are numerous in type, and their inherent resistance to degradation leads to their ubiquitous presence and accumulation in environmental matrices [6,7]. Despite their often low concentrations, the biotoxicity of these pollutants, coupled with their persistence and bioaccumulative potential, presents potential detrimental effects on the environment and biota [8]. As the understanding of the environmental and health impacts of chemical substances deepens, and as environmental detection technologies evolve, an increasing number of these pollutants are being identified. However, there is a noticeable absence of regulatory frameworks overseeing their presence and impact, leading to their collective designation as emerging contaminants (ECs) or emerging pollutants (EPs). It is widely acknowledged that these contaminants, even at low concentrations, can enter biological systems through ingestion, inhalation, or dermal contact [9], with ingestion being the predominant exposure route. Once inside an organism, they accumulate in various tissues and organs [10,11], influencing metabolic pathways, including glucose metabolism. For instance, when mice were exposed to 1.25 mg/kg/d PFOA for 28 days, an elevation in blood glucose levels was observed, accompanied by a reduction in hepatic glucose and glycogen content [12]. Moreover, some studies suggest that certain emerging pollutants might influence host glucose metabolism by altering the composition and function of the gut microbiome. For example, Huang et al. found that when mice fed a high-fat diet were exposed to polystyrene microplastics, there was a marked reduction in the richness and diversity of their gut microbiota, an increase in the relative abundance of Gram-negative bacteria, as well as elevated levels of insulin resistance and pro-inflammatory cytokines [13].

The phenomenon of increasing attention to the impacts of emerging environmental pollutants on the incidence, progression, and complications of diabetes has been the subject of extensive research. A survey of various databases reveals that articles have been published reviewing the changes in glucose metabolism induced by these emerging pollutants [14,15]. However, these articles have primarily focused on the impact of individual pollutants on diabetes and its complications, with a comprehensive review of all emerging pollutants, their presence in the environment, biological exposure pathways, and effects on glucose metabolism remaining unexplored. In addition, studies on the effects of exposure to emerging contaminants that cause disturbances in glucose metabolism through alterations in the structure and composition of the gut microbiota have not been reviewed. Therefore, this review summarizes the effects on glucose metabolism after exposure to the four emerging contaminants and the possible mechanisms, especially through the alteration of glucose metabolism after affecting the gut microbiota, by presenting the four new contaminants. The objective is to furnish a foundation for future research on the influence of these pollutants on diabetes, to enhance public awareness of their potential hazards, and to provide empirical support and scientific underpinning for the management of emerging pollutants and relevant environmental policies.

## 2. Emerging Contaminants (ECs)

Emerging contaminants (ECs) refer to environmental pollutants detectable in the environment and natural ecosystems, posing significant health and environmental risks to both humans and ecological systems. Yet, they remain either unregulated by laws and standards or inadequately addressed [16,17]. When these contaminants are introduced into the environment, their concentrations tend to be low, often rendering their short-term toxic effects unnoticeable. However, their bioaccumulative nature and resistance to degradation result in persistent accumulation. By the time they are detectable, these pollutants have already accrued and posed long-term hazards [18]. The ECs in the environment carry potential dangers to the ecosystem and all living organisms, including humans, such as chronic toxicity, genetic harm, endocrine-disrupting effects, and the “tri-effects” (carcinogenic, teratogenic, and mutagenic effects) [19]. These characteristics have prompted scientists to pay increasing attention to emerging pollutants in the environment.

Surprisingly, over 3000 types of emerging pollutants have been identified globally. Almost every country has detected the presence of these [20]. Their widespread sources and diversity are alarming. China, being a significant producer and consumer of various chemicals, faces challenges as the large-scale production, misuse, and improper management of these chemical substances introduce them into the environment, intensifying environmental pollution concerns [21]. The difficulty in monitoring and the lack of adequate regulatory measures for these contaminants, combined with their persistent and bioaccumulative nature, make them challenging to manage once they enter the environment [18,22,23].

Pharmaceutical factories, plastics, artificial sweeteners, plasticizers, illicit drugs, cleaning products, cosmetics, personal care products, beverages, and packaging are primary sources of these pollutants [24]. They primarily enter biological systems via dietary exposure. Many studies hint that animal-derived food sources are major contributors to many endocrine-disrupting agents. For instance, residues of polychlorinated biphenyl congeners in Crassostrea tulipa (oysters) and Anadara senilis (mussels) were detected at concentrations of 2.95–11.41 mg/kg wet weight and 5.55–6.37 mg/kg wet weight, respectively [25]. The likelihood of exposure to these biphenyls in bivalves is high, with median concentrations exceeding FDA action levels [26]. Wang et al. [27] detected 11 types of perfluorinated compounds in consumer products such as pork tenderloin, pork heart, pork liver, pork kidney, chicken breast, and chicken liver. Every sample contained these compounds, with pork liver having the highest average content of 3.438 ng/g, followed by pork kidney (0.508 ng/g). Additionally, researchers found microplastics in various salts and bottled waters humans consume, with the highest concentrations in sea salt (550–681 particles/kg) and bottled water showing average concentrations of 10.4 particles/L and 325 particles/L for microplastics with sizes >100 μm and <100 μm, respectively [28,29].

**Figure 1:**
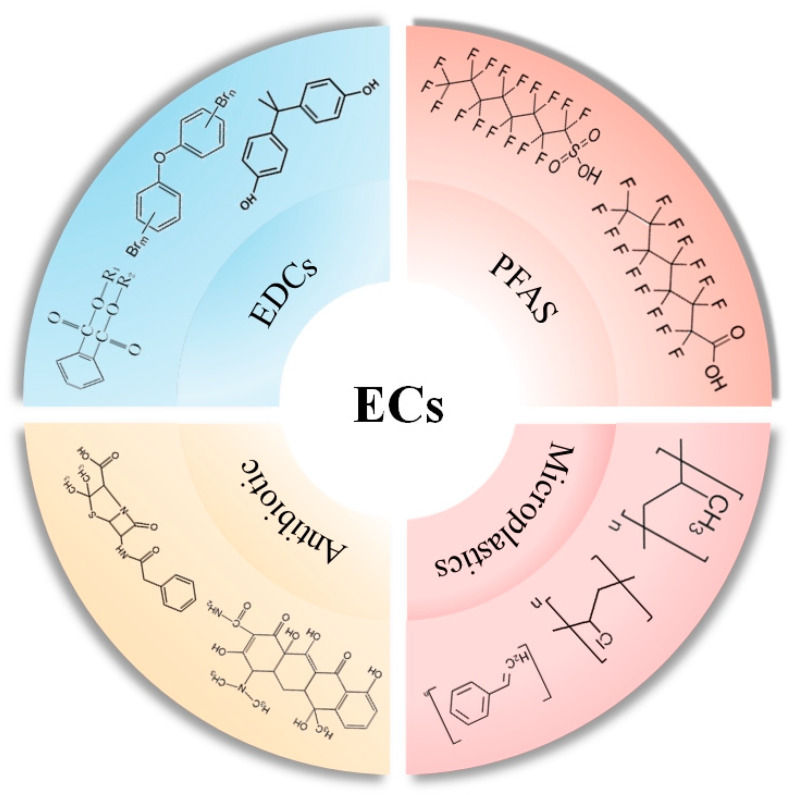
Typical emerging contaminants and representative compounds.

Emerging contaminants closely linked with daily human life can be categorized as biological (e.g., resistant genes, algal toxins), chemical (e.g., novel pesticides, endocrine disruptors, flame retardants, antibiotics, perfluorinated compounds), and physical (e.g., microplastics, nanomaterials) [22,30,31]. Based on publicly available regional or site-specific monitoring data, typical emerging pollutants in China mainly include endocrine disruptors, perfluorinated compounds, microplastics, and antibiotics, all of which are causing severe pollution issues in the air, water, and soil environments [22,30]. Numerous published articles have indicated that the presence of emerging pollutants in the environment increases the risk of diabetes in exposed populations and accelerates the onset and progression of the disease. Moreover, it is well known that the mechanisms of type I diabetes and type II diabetes are different. Type I diabetes is mostly related to genetic factors, while for type II diabetes, lifestyle and exposure to emerging contaminants seem to be more important. This article focuses on these four emerging pollutants, briefly discussing their influence on the onset and progression of diabetes, hoping to offer a scientific foundation for the management and prevention of emerging contaminants (Figure 1).

## 3. Endocrine-Disrupting Chemicals (EDCs)

Endocrine-disrupting chemicals (EDCs) are defined as “an exogenous substance or mixture that alters function(s) of the endocrine system and consequently causes adverse health effects in an intact organism, or its progeny, or (sub) populations” [32]. The most prevalent EDCs include persistent organic pollutants (POPs), phenolic compounds, insecticides, and flame retardants, among others. Due to the frequent use of endocrine-disrupting chemicals (EDCs) in daily life, they are ubiquitously present in the environment. The detection methods for EDCs in the environment primarily include mass spectroscopy, chromatography-based methods, and advanced sensing approaches [33,34,35], such as electrochemical and colorimetric methods. Owing to their rapid, portable, sensitive, and eco-friendly characteristics, sensing approaches are frequently employed to detect EDCs in environmental and food production systems. Some EDCs are lipophilic, and once ingested by humans through the food chain, they accumulate in adipose tissue [36]. An epidemiological study by Miquel Porta discovered that over 85% of study participants had detectable levels of EDCs such as polychlorinated biphenyls (PCBs), DDE, and DDT in their bloodstream. Additionally, the concentration of EDCs in adipose tissue ranged between 92 ng/g and 399 ng/g [37]. Upon entering organisms, EDCs interfere with the natural synthesis, secretion, and elimination of hormones, leading to endocrine imbalances. Such disturbances can result in endocrine disorders, including obesity and diabetes [38] (Table 1).

### 3.1. Persistent Organic Contaminants (POPs)

Persistent organic contaminants (POPs) are toxic chemicals that are highly resistant to environmental degradation and metabolic degradation [39]. Most POPs are hydrophobic and can accumulate continuously in the fat of animals and humans [40], causing significant biological toxicity, such as developmental defects, metabolic diseases, cancer, and even death [41]. The primary POPs in the environment include phthalates (PAEs), polybrominated diphenyl ethers (PBDEs), polychlorinated biphenyls, and dioxins.

Phthalates (PAEs) are frequently introduced into the environment as plasticizers used in various plastic products [42]. Given China’s role as a major producer of PAEs and its lack of effective pollution control measures, the PAE pollution level in Chinese waters is exceptionally high [43]. Moreover, recent studies have shown that when PAEs enter an organism, they can disrupt glucose metabolism and affect blood glucose levels. A case–control study of volunteers from China found that environmental PAEs can be metabolized within the human body, and their metabolites can be excreted in urine. There is a significant positive correlation between these metabolites and fasting blood glucose and glycated hemoglobin levels, thereby interfering with normal glucose metabolism and affecting the development of type II diabetes [44]. Additionally, metabolic pathways closely related to glucose metabolism, such as galactose metabolism, amino acid metabolism, riboflavin metabolism, and pyridoxal metabolism, can also be affected by PAEs, changing their metabolic marker levels. For instance, in a case–control study of volunteers in Tianjin, China, researchers have observed that the metabolic products involved in galactose metabolism in the serum of patients with type II diabetes were significantly elevated and showed a positive correlation with serum PAE levels. Other metabolites involved in amino acid metabolism, riboflavin metabolism, and pyridoxal metabolism also showed significant changes [45]. Similarly to type II diabetes, gestational diabetes also showed a significant correlation with PAE metabolites [46,47]. While there are a growing number of population studies on the relationship between PAEs and diabetes, research on their specific mechanisms is still relatively limited. When rat insulinoma (INS-1) cells were exposed to dibutyl phthalate (DBP), Yang found that exposure at concentrations of 60 μmol/L and 120 μmol/L led to increased cell apoptosis, significant reductions in mitochondrial membrane potential, increased cellular oxidative stress levels, and decreased superoxide dismutase levels. It is speculated that DBP might reduce INS-1 cell insulin synthesis and secretion through mitochondrial apoptosis and oxidative stress pathways [48].

Polybrominated diphenyl ethers (PBDEs) are brominated flame retardants. Since the 1960s, they have been used as flame retardants in commercial and household products (44). Like PAEs, PBDEs continuously accumulate in organisms, affecting their glucose and lipid metabolism. Data from a nested case–control study indicate a significant correlation between brominated biphenyl ethers (BDEs) and gestational diabetes [49]. Furthermore, BDE-153, BDE-154, and BDE-183 all have odds ratios >1, showing a significant positive and inverted U-shaped correlation with diabetes. Similarly, Ongono and colleagues found in a cohort study that dietary exposure to hexabromocyclododecane is positively correlated with type II diabetes and dietary exposure to PBDEs has a positive non-linear relationship with type II diabetes [50]. Liu used mice orally exposed to BDE-153 to explore the potential mechanisms by which brominated flame retardants might affect glucose metabolism. He found that mouse insulin levels showed a dose-dependent relationship with BDE-153 and the expression of PPARγ and AMPKα was disturbed. It is speculated that BDE-153 might interfere with the expression of adipokines and insulin secretion by affecting the expression of PPARγ and AMPKα, leading to metabolic dysregulation [51].

In addition to PAEs and PBDEs, there are numerous other POPs present in the environment, including polychlorinated biphenyls (PCBs) and dioxins. Organisms living within such environments, especially those on a high-fat diet or at risk for diabetes, can experience altered glucose metabolism when exposed to these POPs. Ibrahim et al. [52] fed C57BL/6J mice a high-fat diet containing POPs and observed that, compared to the unexposed group, both the high-fat-diet mice and those on a Western diet exhibited exacerbated manifestations of insulin resistance, visceral obesity, and abnormalities in glucose tolerance. Additionally, it was found that mice in the low-exposure group exhibited better insulin sensitivity and glucose tolerance than those in the high-exposure group. Furthermore, when C57BL/6J mice on a high-fat diet or those with diabetes were exposed to PCBs, they displayed glucose metabolic abnormalities characterized by glucose intolerance, increased gluconeogenesis, elevated tricarboxylic acid cycle flux, hyperinsulinemia, and intensified systemic insulin resistance [41,53]. Interestingly, a study by Nicki A. Baker revealed that while exposure to PCBs induced lipid inflammation and glucose and insulin tolerance impairment in mice on a low-fat diet, the glycemic equilibrium in obese mice remained unaffected unless they underwent weight reduction [54,55].

The continuous accumulation of dioxins, specifically 2,3,7,8-tetrachlorodibenzo-p-dioxin (TCDD), in the environment and their impact on glucose metabolism is gradually gaining attention from scientists. When mice were exposed to environmental TCDD at a dose of 20 ng/kg, the hyperglycemia induced by a high-fat diet and the reduction in plasma insulin levels induced by glucose were intensified. These mice exhibited significantly elevated blood sugar levels, substantial changes in islet endocrine and metabolic pathways, and increased expression of mRNAs encoding the sodium–glucose transporter 1 and glucose transporter 2 in the intestines [56,57]. Kurita’s research yielded similar conclusions: post-TCDD exposure, there was a notable decrease in plasma insulin concentrations in mice, with insulin secretion levels significantly reduced [58]. Furthermore, researchers discovered that when high-fat-diet mice undergoing mating, pregnancy, or lactation were injected with 20 ng/kg TCDD twice a week, these mice experienced accelerated weight gain, faster onset of hyperglycemia, reduced islet levels, and islet shrinkage [59].

The review identified that phthalates, polybrominated diphenyl ethers, polychlorinated biphenyls, and other persistent organic pollutants are widely present in the environment and can enter biological organisms, including humans, through various pathways. Studies have found a significant positive correlation between the concentration of persistent organic pollutants in human serum or urine and fasting blood glucose levels. This phenomenon has been further substantiated in animal and cell experiments, with discussions on potential mechanisms.

### 3.2. Bisphenol A (BPA) and Its Structural Analogs

Bisphenol A (BPA) and its structural analogs (BPS, BPF, BPAF) are synthetically produced and ubiquitously present in the environment, serving as endocrine disruptors. The omnipresence of BPA compounds means that human exposure to this contaminant is inevitable [60]. Once introduced to the human body, BPA can instigate a myriad of detrimental effects, disrupting metabolic processes including lipid metabolism and glycemic regulation [61]. To date, numerous epidemiological studies have scrutinized the association between urinary concentrations of BPA and its metabolites and diabetes, not only pinpointing a robust correlation between BPA and type II diabetes but also identifying it as a risk factor for gestational diabetes. Beyond this, to elucidate the specific role of BPA in the onset and progression of diabetes and to understand its potential mechanisms leading to the disease, numerous animal experiments have been conducted. For instance, when mice on a standard diet were exposed to 5, 50, 500, and 5000 μgBPA/kg for 8 months, they manifested clear hyperglycemia and hypercholesterolemia. Male mice exposed to 5000 μg/kg/d BPA displayed pronounced insulin resistance [62]. Similarly, in mice fed a high-fat diet, research indicated that BPA aggravates the pre-diabetic symptoms induced by such a diet. Intriguingly, while male mice exhibited only impaired glucose tolerance, female mice also demonstrated increased weight and elevated serum insulin levels, among other symptoms [63]. Regarding the glucose intolerance induced by BPA, a study by Moon et al. posits that it might be attributed to altered serum adipocytokine levels and skeletal muscle phosphorylation, subsequently inducing glucose tolerance abnormalities [64].

Bisphenol A, as an endocrine disruptor, is prevalent in the environment. Numerous population studies have also discovered its association with the development of diabetes, and animal experiments have been conducted to investigate the potential mechanisms of bisphenol A in disrupting glucose metabolism.

**Table 1:** Summary of studies related to the effects of POPs on glucose metabolism.

Class	Study Subject	Research Models	Chemical	Toxicity	Reference
Population Studies	General population (250 T2DM and 250 controls)	Case–control study	PAEs	T2DM is significantly positively associated with urinary PAE metabolite concentrations. Urinary mono (2-ethylhexyl) phthalate is significantly and positively correlated with fasting blood glucose levels. Exposure to PAEs is associated with T2DM, fasting blood glucose, and glycosylated hemoglobin levels. Association of exposure to PAEs with T2DM, fasting glucose, and glycosylated hemoglobin may vary among sex, BMI, or age.	[44]
	General population (60 T2DM and 60 controls)	Case–control study	mPAEs, bisphenols	Positive association between urinary mPAEs and T2DM. mPAE exposure may contribute to increased risk of T2DM by interfering with galactose metabolism, amino acid metabolism, and pyrimidine metabolism.	[45]
	First trimester (T1) pregnant women (60 GDM and 90 IGT)	Case–control study	PAEs	PAE metabolites are significantly associated with glucose intolerance and are more strongly associated in some races.	[46]
	First trimester (T1) pregnant women (169 GDM and 169 controls)	Case–control study	PAEs	Urinary MOP, MBZP, MEOHP, and MECPP concentrations in early pregnancy are significantly associated with GDM. Urinary MEOHP concentration was significantly and positively correlated with GDM.	[47]
	First trimester (T1) pregnant women (439 pregnant women)	Nested case–control study	PBDEs	The OR of GDM was significantly positively correlated with the levels of BDE-153 and BDE-183, with an inverted U-shaped correlation with GDM. BDE-153 and -154 were significantly positively correlated with fasting blood glucose, 1 h and 2 h postprandial blood glucose.	[49]
	General population (71,415 women)	Prospective cohort study	Brominated flame retardants (BFRs)	Positive association between dietary HBCD exposure and T2D risk. Positive but non-linear association between dietary exposure to PBDEs and T2D risk.	[50]
Cell Experiments	Rat insulinoma (INS-1) cells	Experimental research	DBP	Cellular insulin synthesis and secretion were significantly reduced. Increased apoptosis rate and significant decrease in mitochondrial membrane potential. As the DBP exposure measure increased, the level of oxidative stress increased and the level of antioxidant index decreased.	[48]
Animal Experiments	Adult male C57BL/6J mice	Experimental research	BDE-153	Induces disorders of glycolipid metabolism in mice. Insulin staining positivity increased in a dose-dependent manner. BDE-153 may interfere with adipokine expression and insulin secretion by affecting PPARγ and AMPKα expression, leading to disorders of glucolipid metabolism.	[51]
	High-fat-diet male mice (C57BL/6J)	Experimental research	POPs	Leading to insulin resistance, visceral obesity, abnormal glucose tolerance. Impaired phosphorylation of insulin secondary Akt and muscle glucose uptake capacity. Better insulin sensitivity and glucose tolerance in mice fed diets with reduced POP concentrations.	[52]
	High-fat-diet male mice (C57BL/6J)	Experimental research	TSP, D_2_O, PCB126	PCB126-induced inflammatory response (including higher levels of serum cytokines and adipose-validated gene expression) observed in high-fat-diet mice. Deterioration of impaired glucose homeostasis characterized by abnormal glucose tolerance, gluconeogenesis, and increased tricarboxylic acid cycle fluxes.	[41]
	High-fat-diet female mice (C57BL/6J)	Experimental research	PCBs	Produces hyperinsulinemia and exacerbates whole-body insulin resistance in obese mice.	[53]
	Low-fat-diet or high-fat-diet female mice (C57BL/6J)	Experimental research	PCB-77, PCB-126	Persistently impaired glucose and insulin tolerance in low-fat-diet mice. Increased expression of TNF-α in fat in PCB-77-treated mice (may contribute to disruption of glucose homeostasis). Glycemic homeostasis was not affected in obese mice in the exposed group but was damaged after weight loss.	[54]
	Low-fat-diet or high-fat-diet male mice	Experimental research	PCB-77	Shows weight gain, lipid inflammation, and impaired glucose tolerance. Increased amount of tumor necrosis factor-α mRNA and impaired glucose homeostasis in PCB-77-exposed mice after weight loss.	[55]
	High-fat-diet mice (C57BL/6J)	Experimental research	TCDD	Accelerated high-fat-diet-induced hyperglycemia and glucose-induced decrease in plasma insulin levels in female mice. Slight increase in male islet area. Abnormal changes in endocrine and metabolic pathways in female pancreatic islets.	[56]
	Male C57BL/6J mice and DBA/2J mice	Experimental research	TCDD	Altered villous structure and nucleoplasmic ratio of intestinal epithelial cells. Significantly increased blood glucose levels in mice. Increased expression of mRNAs encoding sodium–glucose transporter 1 and glucose transporter 2 in the intestine.	[57]
	Male C57BL/6J mice, AhR mice, islets from C57BL/6J mice	Experimental research	TCDD	Significantly reduced plasma insulin concentrations in C57BL/6J mice at 60 and 120 min after glucose stimulation. Significant decrease in insulin secretion levels.	[58]
	High-fat-diet female mice (C57BL/6J)	Experimental research	TCDD	Accelerated weight gain in high-fat-diet mice after exposure to TCDD. Faster onset of hyperglycemia. Decreased plasma insulin levels induced by glucose and shrinkage of pancreatic islets. Low-dose TCDD exposure during pregnancy has long-term adverse effects on metabolic adaptation in HFD-fed offspring mice.	[59]
	Male CD1 mice	Experimental research	BPA	Bisphenol A exposure for 8 months causes hyperglycemia and hypercholesterolemia. The 5000 mg/kg/d BPA exposure group of mice had significantly decreased glucose tolerance.	[62]
	High-fat-diet mice (C57BL/6J)	Experimental research	BPA	Aggravating high-fat-diet-induced pre-diabetes symptoms. Female mice exhibit weight gain, elevated serum insulin levels, impaired glucose tolerance. Male mice show only impaired glucose tolerance.	[63]
	High-fat-diet male mice (C57BL/6J)	Experimental research	BPA	Induces an increase in glucose tolerance. Reducing skeletal muscle phosphorylation by altering serum adipocytokine levels may be one of the mechanisms by which BPA induces abnormal glucose tolerance.	[64]

## 4. Per- and Polyfluoroalkyl Substances (PFASs)

Per- and polyfluoroalkyl substances (PFASs) typically consist of carbon chains ranging from 4 to 14 carbons, complemented by a few functional groups [65]. Due to their intrinsic properties such as thermal stability, hydrophobicity, and oleophobicity, PFASs have found extensive applications in industrial production and consumer goods [66], for instance, non-stick cookware, grease-resistant food packaging, and personal care products [67]. Per- and polyfluoroalkyl substances (PFASs), as novel pollutants, have not been thoroughly researched. Due to their widespread application in consumer products, PFASs are omnipresent in the environment, posing potential threats to both the environment and humans. Perfluorooctane sulfonate (PFOS) and perfluorooctanoic acid (PFOA), the most frequently detected PFASs [68], persist in the environment because of the stability of their carbon–fluorine bonds [69]. The current method employed by the United States Environmental Protection Agency for detecting PFASs in the environment relies on combinations of liquid chromatography and mass spectroscopy [70]. However, due to the high costs and the need for trained specialized laboratory personnel, recent research efforts have focused on developing rapid, portable, and low-cost detection methods. The precise toxicological profile of PFASs remains elusive, but mounting research underscores the potential health risks they pose, inclusive of metabolic disturbances (Table 2).

### 4.1. Perfluorooctane Sulfonate (PFOS)

PFOS, a degradation product among many PFASs, emerges as one of the most scrutinized compounds in the PFAS family. Scientists have ascertained that PFOS not only remains persistent in the environment but also has a notably long half-life of approximately 5.4 years in human serum once ingested, with serum concentrations averaging around 0.05 μg/mL [11,71]. Recently, the connection between PFOS and diabetes has gained research traction.

A growing body of epidemiological evidence associates increased PFAS serum concentrations in humans with elevated fasting glucose, fasting insulin levels, changes in the insulin homeostasis model, and enhanced cellular functions. This compound has been identified not only as a risk factor for gestational diabetes but also as an augmenting agent for type II diabetes susceptibility [72,73,74]. Observations from human population studies are progressively being corroborated by animal experiments. For instance, Sant et al. [75] exposed zebrafish embryos in the blastula stage to 16, 32, 64 Mm PFOS, noting congenital anomalies mirroring the increased risk factors for human diabetes. The embryos and larvae exhibited perturbed pancreatic growth, pancreatic islet malformations, and a U-shaped dose–response relationship with respect to islet size and PFOS exposure. Qin et al. [76] discovered through in vivo and in vitro studies that PFOS exposure stimulates the free-fatty-acid-regulated membrane receptor G protein-coupled receptor 40 in pancreatic β-cells, thereby heightening intracellular calcium levels and insulin secretion. Moreover, insulin secretion was augmented in a concentration-dependent manner upon acute PFOS exposure, with a marked increase observed at concentrations exceeding 50 μM [77]. Intriguingly, Duan et al. [78] yielded contrary findings, indicating that prolonged PFOS exposure (48 h) inhibits glucose-stimulated insulin secretion. Furthermore, in specific cohorts such as pregnant and lactating mice, research has showcased the biological effects of elevated fasting glucose and insulin levels in both F1 juvenile and adult mice due to PFOS exposure. However, insulin resistance and glucose intolerance anomalies were conspicuously observed only in adult mice. Notably, a high-fat diet exacerbated these effects [79].

### 4.2. Perfluorooctanoic Acid (PFOA)

Perfluorooctanoic acid (PFOA) is frequently employed as an emulsifier in the production of polytetrafluoroethylene and fluorinated polymers. Ambient concentrations of PFOA in the air typically range from 0.07–0.9 ng/m^3^ [80], but can spike to 0.12–0.91 μg/m^3^ in the vicinity of fluoropolymer-manufacturing plants [81]. PFOA emissions during the manufacturing process are carried by the wind to adjacent agricultural areas, where they settle in the topsoil layer, eventually seeping downward to the water table [82]. Once introduced into organisms via environmental exposure, PFOA accumulates over time. Ehresmanet reported human serum PFOA concentrations spanning from the detection limit (5 or 10 ng/mL) to 7320 ng/mL [83]. Owing to PFOA’s crucial role in metabolic processes and its newfound potential to influence human glucose metabolism, an escalating number of researchers are probing its implications for diabetes and its hypothesized operational mechanisms.

Numerous epidemiological studies have identified a correlation between serum PFOA levels and the proinsulin-to-insulin ratio, after adjusting for confounding factors. Notably, diabetic subjects exhibit significantly elevated lnPFOA levels compared to their non-diabetic counterparts, and these levels can presage the onset of diabetes [84,85]. Analogous findings have emerged from animal studies concerning PFOA and diabetes. For instance, when Zheng and colleagues administered a dose of 1.25 mg/kg/d of PFOA to mice via gavage for 28 days, the mice in the exposed group, although unchanged in weight, manifested conspicuously elevated fasting blood glucose levels, coupled with decreased hepatic glycogen and glucose content [12]. Similarly, Yan et al. observed heightened insulin sensitivity and glucose tolerance in mice exposed to 5 mg/kg/d PFOA. This was attributed to suppressed hepatic gluconeogenesis, leading to diminished liver glycogen synthesis [86]. While mounting research is spotlighting the influence of PFOA on blood glucose levels, the underpinning mechanisms remain only partially elucidated. In an investigation by He [87] on the potential impact of PFOA on the functionality of pancreatic β-cells in mice, it was discerned that at a dose of 500µM, PFOA stimulates β-cell apoptosis. Moreover, even lower doses of PFOA resulted in diminished insulin secretion upon glucose stimulation and a pronounced upregulation of endoplasmic-reticulum-stress-related gene expression.

The review finds that exposure to perfluorinated compounds leads to increased fasting blood glucose levels and disrupted glucose metabolism in mice, along with alterations in the morphology, size, and length of the islets, thereby impacting insulin secretion. However, the underlying mechanisms of these effects have yet to be fully elucidated.

**Table 2:** Summary of studies related to the effects of PFAS on glucose metabolism.

Class	Study Population	Research Models	Chemical	Toxicity	Reference
Population Studies	General population (474 adolescents and 969 Adults)	Cross-sectional study	PFNA, PFOS, PFOA, etc.	Hyperglycemia is associated with elevated PFNA concentrations. Elevated serum PFNA concentrations are negatively associated with the prevalence of metabolic syndrome. Elevated serum PFOS concentrations correlate with elevated insulin, assessment of insulin resistance in a homeostasis model, and cellular function.	[72]
	General population (1045 adults)	Cross-sectional study	PFOS and PFOA	Fasting blood glucose, fasting insulin, and pancreatic β-cell function correlate with increased concentrations of Br-PFOS. Long-term exposure to PFAS isomers is associated with impaired glucose homeostasis and may increase the prevalence of type II diabetes mellitus among Chinese adults.	[73]
	Pregnant women (171 GDM and 169 controls)	Cross-sectional study	PFOS, PFOA, PFNA, etc.	Concentrations of PFAS congeners positively correlate with fasting glucose, 1 h and 2 h blood glucose after glucose tolerance test, and sustained glucose results on glycated hemoglobin. Risk of gestational diabetes and blood glucose levels increase significantly with increasing concentrations of PFOS mixtures.	[74]
	General population (1016 men and women aged 70 years)	Prospective cohort study	PFOA, PFNA	After adjusting for confounders, PFNA and PFOA showed a significant non-linear relationship with diabetes mellitus. PFOA is associated with the insulinogen/insulin ratio but not with markers of insulin resistance.	[84]
	General population (100 participants)	Prospective cohort study	PFOA, PFOS	Log-transformed PFOA and log-transformed PFOS were significantly higher in diabetic patients than in non-diabetic patients. LnPFOA significantly predicts diabetes.	[85]
Cell Experiments	GPR40-KO C57BL/6, C57BL/6 mice and mouse islet β-cells	Experimental research	PFOS	Stimulation of insulin secretion and intracellular calcium levels by activation of the GPR40, an important free-fatty-acid-regulated membrane receptor on islet β-cells.	[76]
	Beta-TC-6 pancreatic cells	Experimental research	PFOS	Acute exposure to PFOS stimulates insulin secretion and increases intracellular calcium ion concentrations. PFOS stimulates insulin secretion at least in part by GPR40.	[77]
	Mouse pancreatic β-cells	Experimental research	PFOS	Continuous exposure to PFOS inhibits glucose-stimulated insulin secretion but has no significant effect on insulin gene expression. SIRT1 activators and UCP2 inhibitors partially reverse PFOS-induced impairment of insulin secretion.	[78]
	Mouse pancreatic β-cell line (MIN6 cells)	Experimental research	PFOA	Time- and dose-dependent inhibition of cell viability. A high dose (500 μM) of PFOA promoted β-cell apoptosis, and a low dose (300 μM) had no effect on cell survival. Low doses also reduce glucose-stimulated insulin secretion. Endoplasmic-reticulum-stress-related proteins were significantly increased, and inhibition of TRIM3 expression significantly protected MIN6 cells from PFOA-induced defective insulin secretion and apoptosis by ameliorating endoplasmic reticulum stress.	[87]
Animal Experiments	Zebrafish (*Danio rerio*) embryos	Experimental research	PFOS	Abnormal islet morphology. Islet size and pancreas length showed a U-shaped dose–response relationship with PFOS. Embryonic PFOS exposure can disrupt pancreatic organogenesis in a manner that mimics human genetic defects and predisposes individuals to diabetes mellitus.	[75]
	Pregnant mice and offspring	Experimental research	PFOS	Elevated fasting glucose and insulin levels in F1 offspring juveniles and adults. Insulin resistance and abnormal glucose tolerance were only evident in F1 adults.	[79]
	Adult male Balb/c mice	Experimental research	PFOA	No significant change in body weight in the exposed group of mice. Fasting blood glucose levels were elevated and glycogen and glucose levels in the liver were reduced. Increased glucose production capacity.	[12]
	Male Balb/c	Experimental research	PFOA	Induced higher insulin sensitivity and glucose tolerance. Reduced hepatic glycogen synthesis, which may be due to inhibition of gluconeogenesis. Changes in levels of centrally circulating proteins (including proteins that may be associated with diabetes and liver disease).	[86]

## 5. Microplastics

Microplastics refer to plastic particles with a diameter of ≤5 mm. Traditional methods for detecting microplastics in the environment include visual identification or microscopic observation, Fourier transform infrared spectroscopy, thermal pyrolysis, and Raman spectroscopy. However, the diverse sources and compositions of environmental microplastics, along with the presence of numerous impurities, render these conventional detection methods inadequate for comprehensive microplastic detection [88]. With increasing interest in the toxicity of microplastics and advancements in detection technology, more sensitive and high-performance detection techniques are being developed, such as a variety of remote sensing techniques including polarized light optical microscopy (PLM), atomic force microscopy, and hybrid combinations of these techniques [89]. Humans and other organisms are exposed to environmental microplastics through ingestion, inhalation, and dermal contact [90]. Furthermore, oral exposure has been reported as the primary route of microplastic exposure. Kumar et al. [91], in their review, mention seafood, beer, table salt, bottled mineral water, and milk as the main pathways for microplastics to enter the human body. Once internalized, microplastics accumulate within tissues and organs, leading to histopathological alterations and cytotoxic responses [6,92]. For instance, a study by Lu et al. on zebrafish found that exposure to microplastics first leads to accumulation in liver tissues, causing inflammation and lipid accumulation, and disrupting lipid and energy metabolism, leading to metabolic changes [93]. Cortés et al.’s cellular experiments also found that microplastics induce the production of a significant amount of ROS in Caco-2 cells, thereby increasing cytotoxicity [94]. The same conclusion was reached in cell experiments with T98G and HeLa [95]. Additionally, immune responses and changes in the structure and composition of the gut microbiota induced by microplastic exposure have also been increasingly identified [91]. It is well-established that gut microbiota dysbiosis, inflammatory reactions, oxidative stress, and changes in innate immune responses—all consequences of microplastic exposure—are major pathophysiological factors for insulin resistance. Consequently, scientists posit a strong link between microplastic exposure and insulin resistance, necessitating comprehensive research and elucidation. However, current investigations in this area remain limited (Table 3).

Studies have identified correlations between changes in blood glucose levels and insulin resistance caused by microplastic exposure, specifically noting connections to gut microbiota disruption, inflammation, and oxidative stress. Huang et al. [13] exposed mice on a high-fat diet to polystyrene microplastics of sizes 5, 50, 100, and 200 μm. The mice displayed insulin resistance accompanied by elevated levels of plasma lipopolysaccharides and pro-inflammatory cytokines (tumor necrosis factor and interleukin-1β). A reduction in gut microbiota richness and diversity was also observed, particularly with an increased relative abundance of Gram-negative bacteria. Based on these findings, scientists hypothesize that insulin resistance triggered by microplastics might be due to tissue accumulation and microbiota-induced inflammatory responses, thereby inhibiting the insulin signaling pathway. Takuro Okamura also demonstrated that mice exposed to microplastics showed elevated blood glucose levels and deposition of microplastics in the gut mucosa, resulting in an increase in intrinsic inflammatory cells and a reduction in anti-inflammatory cells [96]. Additionally, the insulin resistance and elevated blood glucose levels induced by microplastics might be associated with high levels of reactive oxygen species (ROS) in mice exposed to 5 mg/kg and 15 mg/kg, which potentially disrupt the PI3K/Art pathway related to glucose metabolism [97]. In another study, beyond increasing oxidative stress, glucose tolerance, and insulin resistance, 30 mg/kg/d microplastic exposure also led to decreased phosphorylation levels of AKT and GSK3β [98]. AKT agonists can effectively alleviate oxidative stress, elevated blood glucose levels, and insulin resistance, suggesting that part of the diabetes mechanism induced by microplastics might be related to AKT/GSK3β phosphorylation. Furthermore, research has identified that reduced cortisol levels in mice exposed to 55 μg/d microplastics might interfere with insulin secretion, thereby inducing insulin resistance [99]. A review of microplastics reveals that current research on the relationship between microplastics and glucose metabolism is relatively scarce. Studies have found that exposure to microplastics can affect an organism’s glucose metabolism and the development of diabetes through inflammatory responses, oxidative stress, and disruption of the composition and structure of the gut microbiota.

**Table 3:** Summary of studies related to the effects of MPs on glucose metabolism.

Class	Study Population	Microplastics	Toxicity	Reference
Animal Experiments	Five-week-old high -fat-diet male mice (Mus musculus, ICR)	Polystyrene (5, 50, 100, and 200 μm)	Mice in both the normal and high-fat-diet groups exhibited insulin resistance with elevated plasma lipopolysaccharide and pro-inflammatory cytokines (tumor necrosis factor and interleukin-1β). Significant decrease in the abundance and diversity of intestinal microbiota and increase in the relative abundance of Gram-negative bacteria (*Pseudobacillaceae* and *Enterobacteriaceae*). Small particles of microplastics (5 μm) aggregated in the liver, kidney, and blood vessels in mice. Inhibition of insulin signaling pathway in liver (inhibited IRS1 and reduced PI3K expression). The mechanism may stimulate inflammatory responses and inhibit insulin signaling pathways by regulating intestinal microbiota and polystyrene accumulation in tissues.	[13]
	High-fat-diet male mice (C57BL/6)	Polystyrene (0.45–0.53 μm)	High-fat-diet mice exposed to polystyrene microplastics had higher blood glucose, lipid concentrations, and non-alcoholic fatty liver disease scores and more inflammatory cells and fewer anti-inflammatory cells in the lamina propria than those that were not. High-fat diet mice exposed to polystyrene microplastics had significantly higher expression of genes associated with inflammation, long-chain fatty acid transporter proteins, and Na+ glucose cotransporter proteins than those that were not. *Desulfovibrio* genes are significantly enriched in the intestines.	[96]
	Mice	Polystyrene nanoplastics	Oral exposure to nanoplastics can cause organ damage, mainly in liver function and lipid metabolism. Chronic exposure significantly increased blood glucose levels and ROS levels but did not affect plasma insulin secretion. High levels of ROS interfere with the PI3K/Art pathway, leading to insulin resistance and elevated blood glucose.	[97]
	High-fat-diet male mice (C57BL/6)	Polystyrene (80 nm)	Exposure to 30 mg/kg/d of microplastics alone significantly increased blood glucose, glucose tolerance, and insulin resistance. Exposure of high-fat-diet mice to polystyrene microplastics significantly exacerbates oxidative stress, glucose tolerance, and insulin resistance and induces liver and pancreas damage. Polystyrene microplastics exacerbate type II diabetes by an underlying mechanism in part related to AKT/GSPK3β phosphorylation (associated with ROS).	[98]
	ICR mice	Polystyrene (1 μm)	Mice exposed to microplastics for a week suffered significant liver damage and oxidative stress, disturbed liver–gut axis, and increased risk of insulin resistance. Oxidative stress occurred in the liver. When exposed to microplastics for two weeks, lower cortisol in the liver may interfere with insulin secretion and induce insulin resistance. Disturbed gut microbiota. Fasting blood glucose, fasting insulin, and HOMA-IR levels were significantly elevated after microplastics exposure.	[99]

## 6. Antibiotics

Besides the aforementioned emerging pollutants, antibiotics have also been identified as a significant new class of pollutants, extensively present in the environment and water bodies. By the late 1990s, antibiotics had become widely used in medicine and established as pillars of modern medical practice. The consumption of antibiotics has seen a steady increase, with global consumption growing by 39% between 2000 to 2015. Particularly, antibiotic consumption in low-income countries surged by 77% during the same period [100]. In 2011, the global human utilization of antibiotics was estimated at 70 billion, equivalent to an annual consumption rate of 10 per individual [101]. Subsequently, these antibiotics, or their metabolites, enter the environment through human and animal urine and feces, ultimately persisting in soil and aquatic environments [102]. Current detection of antibiotics mainly relies on instrumental analysis, which is highly sensitive. However, due to high costs and laborious pre-treatment, traditional instrumental analysis methods are no longer sufficient for the growing number of samples. Therefore, the development of rapid, high-throughput, and low-cost detection methods is essential. Current methods for detecting antibiotic residues include the microbial method, electrochemical method, high-performance liquid chromatography, liquid mass spectrometry, fluorescence method, Raman spectroscopy, etc. [103]. Scientists have detected various antibiotics, such as amoxicillin, clindamycin, and ciprofloxacin, in the inlet and outlet water of wastewater treatment plants. Furthermore, the highest concentrations of triclocarban and triclosan detected in Indian aquatic environments have reached 5860 ng/L [104], indicating the non-negligible potential hazards of residual antibiotics in the environment. It is widely recognized that antibiotic intake can impact the structure, composition, and function of gut microbiota. Moreover, alterations in the gut microbiota have been closely linked with the onset and progression of diabetes [105]. Consequently, there is mounting concern within the scientific community regarding the relationship between antibiotic consumption and diabetes.

A significant body of research has been conducted to investigate the association between antibiotics and diabetes. For instance, several studies have shown that mice on a high-fat diet or those modeled for diabetes, when treated with antibiotics, exhibited reduced levels of endotoxins in plasma and inflammatory factors in adipose tissue. Healthy mice, on the other hand, displayed beneficial effects on glucose metabolism, including reduced fasting blood glucose and decreased area under the glucose tolerance curve [106,107]. Interestingly, another prospective cohort study revealed that patients treated with antibiotics for durations ranging from twenty-five days to two months, or more than two months, saw their risks for type II diabetes increase by 23% and 20%, respectively [108]. In light of this intriguing observation, Fu et al. [109] studied the impact of antibiotic treatment on blood glucose changes in db/db mice. They found that the effects of antibiotics on blood glucose exhibit both immediate and delayed responses: compared to the control group, mice treated with antibiotics for 12 days showed significant declines in body weight and blood glucose levels. However, 24 days post-treatment, these mice experienced weight gains that even surpassed those of the control group, along with elevated levels of plasma and liver total cholesterol and an increase in liver weight. Research on the relationship between antibiotics and diabetes is already quite abundant, with a relatively comprehensive understanding of the relationship between antibiotics and the development of diabetes. It should not be neglected that the timing of antibiotic exposure is also very important and should not be overlooked, such as in pregnant mothers, infants, and adulthood. A comprehensive review of the relationship between antibiotics and diabetes has been presented in an article by Fenneman and will not be further elaborated upon here [110].

## 7. Role of Gut Microbiota

In recent years, the gut microbiota has garnered unprecedented attention. An increasing corpus of evidence underscores its fundamental role in the digestion of polysaccharides, the biosynthesis of vitamins, and other essential nutrients. This microbial community is inextricably linked with human health [111,112,113]. Undoubtedly, acting as a novel organ, the gut microbiota functions optimally. However, disruptions in its composition and structure due to external substances can have implications for disease onset and progression [114,115,116]. Particularly noteworthy are recent studies highlighting how exposure to emergent environmental contaminants can destabilize the gut microbiota, leading to adverse health effects, including disorders in glucose metabolism [99,117,118,119] (Table 4).

The current scientific discourse is replete with research focusing on the implications of perfluorinated compound exposure on diabetes via its perturbative effects on gut microbiota. Lai et al. [120] embarked on a study exploring the impacts of dietary PFOS exposure on the gut microbiota of adult mice and scrutinized the consequent changes in induced metabolic functions. Their findings delineated a marked increase in the abundance of *Turicibacterales* and *Allobaculum* in the exposed group of mice, juxtaposed with a significant decline in *B. acidifaciens*. Moreover, the researchers discerned that mice exposed to 3 μg/g/day PFOS exhibited a precocious decline in blood glucose levels after oral glucose ingestion. The area under the curve manifested a conspicuous reduction, and after intraperitoneal insulin injection, these mice’s blood glucose levels were markedly lower than those in the control group. Delving deeper into another emergent pollutant, microplastics, it has been discerned that, post-exposure, it can influence the onset and trajectory of diabetes via various mechanisms, with the resultant disruption in gut microbiota being non-trivial. Using mice as model organisms, investigations into the aftermath of microplastics exposure on the gut microbiota were conducted. The outcomes indicated that, post-exposure, there was a perturbation in the gut–liver axis of the mice, a pronounced reduction in gut microbiota diversity, diminished richness of *Bacteroidetes* and *Verrucomicrobia*, and an increased abundance of *Firmicute*, *Deferribacteres*, and *Actinobacteria*. Concurrently, scientists observed that microplastic-exposed mice presented elevated fasting blood glucose and insulin levels [99]. These findings echo the results from Huang et al., who, in addition to observing reduced microbial richness, also noted an increased relative abundance of Gram-negative bacteria within the mice [13]. It is common knowledge that antibiotics have had a longstanding history of use, targeting pathogenic strains within the microbiota. Yet, their administration might also inadvertently impact other microbial communities, resulting in a decrease in the host’s short-chain fatty acid content. This, in turn, might disrupt metabolic processes and energy assimilation, potentially influencing the onset and progression of diabetes [117,121]. Currently, articles published on the topic of new pollutants and their impact on glucose metabolism through the influence on the structure and composition of the gut microbiota are relatively few, with most studies focusing on perfluorinated compounds and microplastics. Additionally, research in this area remains significantly under-developed. The changes in the composition and structure of the gut microbiota after exposure to new pollutants, alterations in their metabolic pathways and metabolites, and the specific mechanisms of their impact on blood sugar still require more in-depth investigation.

**Table 4:** Summary of studies related to the effects of emerging contaminants on glucose-metabolism-associated gut microbiota.

Class	Species	Chemical	Changes in Intestinal Microbiota		Reference
Animal Experiments	Female CD-1 mice	PFOS	Significant changes in the abundance of metabolizing bacteria associated with phyla *Firmicutes*, *Bacteroidetes*, *Proteobacteria*, and *Cyanobacteria* in mouse gut microbiota after PFOS exposure. A significant increase in the number of *Turicibacterales* and *Allobaculum* and a significant decrease in the number of *B. acidifaciens* were observed in the exposure mice. Disruption of intestinal metabolism leads to significant changes in the metabolism of amino acids, methane, and short-chain fatty acids in mice, and these metabolites are thought to be associated with inflammation and metabolic functions.	Disturbances in fat and glucose metabolism. The OGTT experiment showed that PFOS exposure caused abnormalities in glucose tolerance, which were more pronounced in the high-exposure group. After insulin treatment, the blood glucose of mice in the high-dose exposure group was significantly lower than that of mice in the control group. Pyruvate conversion was significantly lower in exposed mice compared to control mice.	[120]
	ICR mice	Polystyrene microplastics (1 μm)	The gut microbiota of mice in the exposure group was significantly altered and the diversity was significantly reduced. At the phylum level, the relative abundance of *Bacteroidetes* and *Verrucomicrobia* was significantly reduced in exposure group mice, whereas the relative abundance of *Firmicute*, *Deferribacteres*, and *Actinobacteria* was increased. At the genus level, the relative abundance of *Lactobacillus* and *Bifidobacterium* increased in exposure groups, while the relative abundance of *Oscillospira*, *Akkermansia*, and *Desulfovibrio* decreased.	Plasma cortisol levels are elevated and non-hepatic glycogen utilization is inhibited. Elevated plasma insulin levels and increased levels of hepatic gluconeogenesis.	[99]
	Male ICR mice	Polystyrene microplastics (5, 50, 100, 200 μm)	Significant decrease in the abundance and diversity of gut microbiota and increase in the relative abundance of Gram-negative bacteria. At the phylum level, the relative abundance of Bacteroidetes increased and the relative abundance of Firmicutes decreased. At the family level, the relative abundance of *Muribaculaceae* and *Helicobacteraceae* decreased in exposure groups, while the relative abundance of *Prevotellaceae*, *Enterobacteriaceae*, *Desulfovibrionaceae*, and *Rikenellaceae* increased.	Inhibition of insulin signaling pathway and reduced expression of IRS1 and PI3K in mouse liver. Increased fasting blood glucose and insulin levels in exposure group mice compared to the control mice. Significantly increased area under the curve of OGTT and ITT curve and elevated HOMA-IR in microplastic-exposed group of mice.	[13]
	NOD/Shiltj mice	Antibiotics	Reduced gut microbiota abundance in exposure group of mice.	Receiving pulsed therapeutic antibiotics early in life accelerates the development of type I diabetes and islet disease.	[117]

## 8. Conclusions

**Figure 2:**
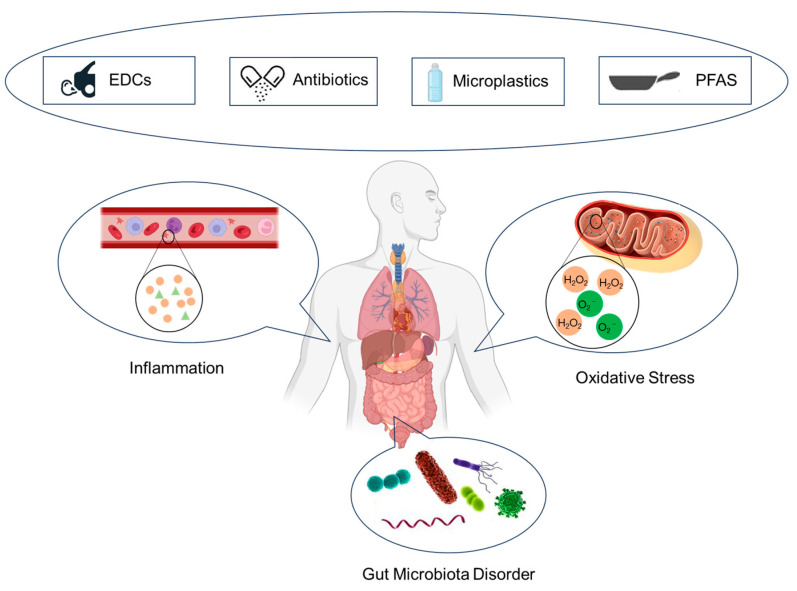
Mechanisms of glucose metabolism disturbance induced by emerging contaminants.

Emerging pollutants, characteristically under-monitored and under-regulated in the environmental sphere, harbor potential, both known and speculative, adverse implications for ecological systems and human health. However, the corpus of research delineating the toxicological nexus between emerging contaminants and glucose metabolic processes remains markedly under-developed. This review aims to intricately weave together four distinct classes of emerging pollutants with glucose metabolism. It methodically dissects and analyzes the toxicological profiles and underlying mechanisms of persistent organic pollutants, perfluorinated compounds, microplastics, and antibiotics, drawing upon a synthesis of empirical evidence from animal model studies and epidemiological research (Figure 2). Overall, EDCs, PFAS, microplastics, and antibiotics cause disturbances in glucose metabolism and accelerate diabetes mellitus. Most of the descriptions of the mechanisms in the currently published articles focus on the effects on glucose metabolism through inflammatory responses, oxidative stress, and disturbances in the gut microbiota. The elucidation provided herein seeks not only to augment the current understanding of the deleterious effects of these emerging pollutants on glucose metabolism but also to catalyze a paradigm shift in the toxicological examination of emerging environmental contaminants.

## 9. Future Research Directions

With the continual evolution of technology and the deepening understanding of contaminants by scientists, an increasing number of novel environmental contaminants are being detected. The persistent accumulation of these contaminants in the environment, coupled with their multifarious exposure pathways, endangers ecosystems and the organisms residing therein, thus progressively capturing the attention of the scientific community. The repercussions of these environmental contaminants, particularly on populations with diabetes or those on high-fat diets, are of heightened concern. Nonetheless, our comprehension in this domain remains somewhat limited:

i. The majority of current epidemiological studies focus on the relationship between the concentration of new pollutants in the serum or urine of the general population and fasting blood sugar, insulin, and glycated hemoglobin concentrations, with some studies involving changes in metabolic pathways related to glucose metabolism and their metabolites. However, studies on the impact of occupational exposure on diabetes, the specific exposure situations of new pollutants in the diet, the correlation with diabetes, and the impact of different geographical locations are relatively scarce. Diet is likely to be a very important exposure, yet rarely assessed in human studies, or when assessed, by questionnaires, often inaccurate. Quantifying the contribution of the human diet by multitargeted metabolomics of food and microbiota-derived metabolites may provide some clues. Therefore, more in-depth and targeted research is still needed to explore the impact of different factors on the development of diabetes.
ii. The current research primarily focuses on the effects of novel environmental contaminants and their exposure on glucose metabolism in human populations, with fewer studies being directed towards animal models. Thus, there remains a pressing need for comprehensive studies to elucidate the specific mechanisms underlying the impact of these contaminants on diabetic or high-fat-diet populations, as well as the potential health outcomes from long-term low exposure.
iii. Additionally, factors influencing the toxic effects of these novel contaminants, such as dose–response relationships, exposure frequency, gender disparities, and attributes like the type and size of the contaminants, have yet to be thoroughly investigated. Hence, there is an urgent need for more in-depth research into the toxicity of these new contaminants, factors modulating their toxicity levels, and their potential hazards. Such insights would furnish policymakers with a robust scientific foundation, aiding in the resolution of environmental challenges and the safeguarding of human health.
iv. It is widely acknowledged that diabetes is influenced not only by genetic, environmental, and lifestyle factors but also by the structure and composition of the gut microbiota. However, current research on the interrelation between novel contaminants, gut microbiota, and diabetes is relatively scant. Consequently, determining whether exposure to these new contaminants might influence glucose metabolism by altering the gut microbiota’s structure and composition calls for relentless effort and exploration by researchers.

## Data Availability

Not applicable.
